# A Systematic Review of Tactile Functioning in Blind Children From a Clinical Perspective

**DOI:** 10.7759/cureus.51180

**Published:** 2023-12-27

**Authors:** Zeinh H Fardan, Shahad H Jabali, Lujain A Alasmre, Hind A Alasmre, Asia A Alsagri, Raghad Z Abuthyab, Aqeelah M Aldarorah, Hussein Almahdi, Yousef Yahya Y Al Qassim

**Affiliations:** 1 Department of Pediatrics, King Khalid University, Abha, SAU; 2 College of Medicine, King Khalid University, Abha, SAU; 3 College of Medicine and Surgery, King Khalid University, Abha, SAU; 4 Department of Medicine and Surgery, Imam Mohammad Ibn Saud Islamic University (IMSIU), Riyadh, SAU; 5 Department of General Practice, Medical University of Lodz, Lodz, POL

**Keywords:** systematic review, children, tactile functioning, visual impairment, blind

## Abstract

In the literature, there is a lot of variation in how well visually impaired youngsters can distinguish between tactile images. This systematic review investigated tactile functioning approaches' clinical perspective on blind children. PubMed, SCOPUS, Web of Science, Science Direct, and Cochrane Library were systematically searched to include the relevant literature. Rayyan QCRI was used throughout this systematic approach. The study included nine studies with a total of 394 children, 246 (62.4%) were males, and 148 (37.6%) were females. Textured graphical objects, images, drawings, and illustrations were used as stimuli to test tactile functioning in blind children. The findings of this comprehensive review showed that tactile stimuli for blind children were most effective in the form of textured images, words, and objects. It has been shown that the complexity, familiarity, and category information all influence how easy or challenging picture recognition is. Blind people can effectively use pictorial displays, but when foreshortening is used in complex representations of three-dimensional objects, they may benefit from instruction.

## Introduction and background

Blindness is defined as visual acuity of less than 3/60 or corresponding visual field loss in the better eye with the best possible correction [1]. For people who were born blind, tactile images have a lot of potential applications [2]. Like maps, they might provide relevant spatial information. In addition, since the majority of personal computer operating systems rely on visuals, tangible graphics may be advantageous for human-computer interfaces. Nevertheless, we now have a limited understanding of how two-dimensional imaging could convey spatial configurations for touch and much less understanding of potential constraints in blind people as a result of insufficient experience. We do know that blind persons are capable of understanding the viewpoint of other viewers while analyzing raised-line images of a model house [3] or tangible images of a variety of objects on a tabletop [4].

For a very long time, philosophers and psychologists have debated whether sensory deprivation enhances the remaining senses [5]. However, it has been asserted that vision is required to calibrate the other senses' experience of space, particularly spatial perception [6]. The processing of the remaining senses could, therefore, also suffer from blindness. Over the past few decades, an increasing number of carefully designed experimental studies have shown that blind people engage in compensatory behaviour in a variety of cognitive tasks. Additionally, the advancement of non-invasive brain imaging methods has provided information on the neurological underpinnings of this compensatory behaviour in humans. The emphasis of the next section's review of these findings will be auditory processing. However, most environmental events offer many sensory systems simultaneous input. One issue under investigation is whether the ability to integrate information from several modalities is innate or whether it progressively emerges and is influenced by experience. The study of whether and how multisensory functions are molded by experience can be done using blind people as a paradigm. We will thus discuss findings on auditory-tactile interactions in blind persons in the second section of our study [7].

It is not necessary to have prior visual experience in order to perceive images, although it is undoubtedly challenging for participants to comprehend tangible images when the task is new. Additionally, tactile images can be created and comprehended by blind persons. The experiments conducted yet highlight the value of visual experience, but it is unclear at this time whether the interpretation of physical graphics would be constrained in any way by a lack of visual experience. Posing limitations, though, with scant supporting data is counterproductive. Congenitally blind persons may beat blindfolded sighted participants in various picture-perception tasks, which further challenges the idea that any group of people has intrinsic limitations [8]. This systematic review investigated the clinical perspective of tactile functioning approaches to blind children.

## Review

Methodology

This systematic review was conducted in accordance with accepted standards (Preferred Reporting Items for Systematic Reviews and Meta-Analyses, PRISMA) [9].

Study design and duration

This was a systematic review conducted between August and September 2023.

Search strategy

A thorough search of five major databases, including PubMed, SCOPUS, Web of Science, Science Direct, and Cochrane Library, was done to find the relevant literature. We restricted our search to English and considered each database's unique requirements. The following keywords were converted into PubMed Mesh terms and used to find the relevant studies; "Tactile," "Blind," "Visual impairment," "Visually deprived," "Children," and "Pediatric." The Boolean operators "OR" and "AND" matched the required keywords. Publications with full English text, available free articles, and human trials were among the search results.

Selection criteria

Study designs that investigated tactile functioning approaches' clinical perspective on blind children were included in this study, participants younger than 18 years, studies conducted between 2000-2023, English studies, and free accessible articles were included.

Data extraction

The search strategy's output was checked for duplication using Rayyan (QCRI) [10]. The researchers evaluated the relevance of the titles and abstracts by modifying the combined search results using a set of inclusion/exclusion criteria. The reviewers carefully examined each paper that met the criteria for inclusion. Techniques for resolving disputes were covered by the authors. With the use of a previously created data extraction form, the authorized study was uploaded. The authors extracted data about the study titles, authors, study year, country, participants, gender, Objectives, Stimuli, and main outcomes. A separate sheet was created for the risk of bias assessment.

Strategy for data synthesis

To give a qualitative analysis of the findings and study components, summary tables were made utilizing data from relevant research. Once the data for the systematic review were retrieved, the most efficient way to use the data from the included study articles was chosen.

Risk of bias assessment

The ROBINS-I risk of bias assessment method for non-randomized trials of treatments was used to assess the quality of the included studies [11]. The seven topics that were assessed included confounding, participant selection for the study, classification of interventions, deviations from intended interventions, missing data, assessment of outcomes, and selection of the reported result.

Results

Search Results

**Figure 1:**
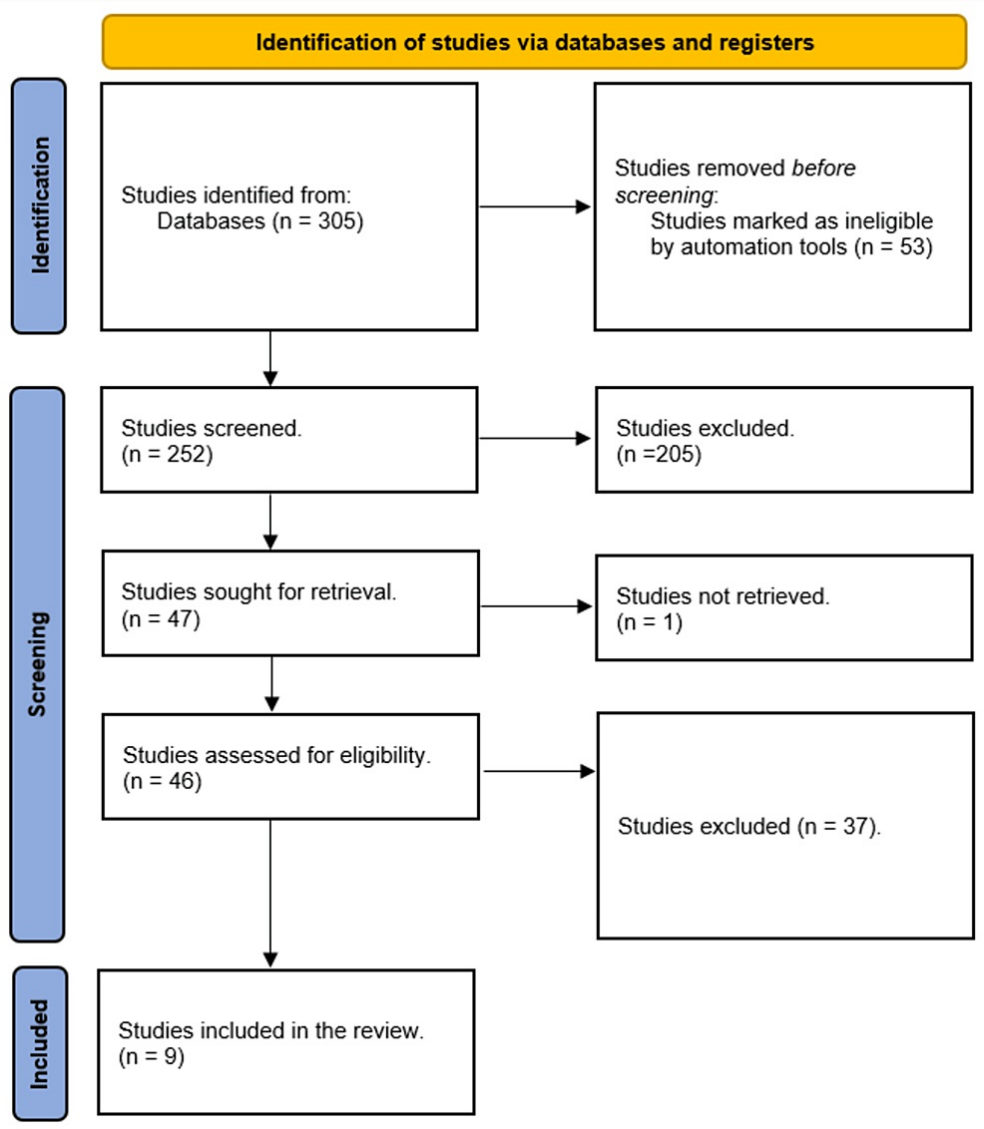
PRISMA flowchart summarizes the study selection process.

A total of 305 study articles resulted from the systematic search, and 53 duplicates were deleted. Title and abstract screening were conducted on 252 studies, and 205 studies were excluded. Forty-seven reports were sought for retrieval, and only one article was not retrieved. Finally, 46 studies were screened for full-text assessment; 22 were excluded for wrong study outcomes, and 15 for the wrong population type. Nine study articles were included in this systematic review. A summary of the study selection process is presented in Figure 1.

Characteristics of the Included Studies

Table 1 presents the sociodemographic characteristics of the included study articles. Our results included nine studies [12-20], with a total of 394 children, and 246 (62.4%) were males. Three studies were conducted in France [12-14], two in the USA [17, 19], one in the Netherlands [15], one in Canada [16], one in China [18], and one in Portugal [20]. Eight studies were case-control studies [12-14, 16-20], and one was a clinical perspective [15].

**Table 1:** Sociodemographic characteristics of the included participants.

Study	Study design	Country	Participants	Mean age (years)	Males (%)
Mascle et al., 2022 [12]	Case-control	France	54	7.5	26 (48.1)
Vinter et al., 2020 [13]	Case-control	France	72	7	32 (44.4)
Theurel et al., 2013 [14]	Case-control	France	23	10.4±3.4	12 (52.2)
Withagen et al., 2010 [15]	A Clinical Perspective	Netherlands	48	birth- 12	22 (45.8)
D'angiulli and Waraich, 2002 [16]	Case-control	Canada	7	NM	3 (48.2)
Ortiz Alonso et al., 2015 [17]	Case-control	USA	24	8-11 (range)	13 (54.2)
Shen et al., 2022 [18]	Case-control	China	132	12.8	118 (89.4)
Ortiz et al., 2019 [19]	Case-control	USA	24	9.9±1.1	14 (58.3)
Alexandre et al., 2012 [20]	Case-control	Portugal	10	6.8±0.7	6 (60)

Table 2 presents the clinical characteristics. Textured graphical objects, images, drawings, and illustrations were used as stimuli to test tactile functioning in blind children [12-14, 16, 19]. Textures facilitate picture recognition regardless of user level, and early and frequent usage of tactile material increases haptic competency. Practical consequences for tactile picture design are examined [14]. For six months, subjects attended twice-daily training sessions five days per week. Children who are blind are better than controls at learning to recognize sensory substitution device (SSD)-delivered letters, and they have a larger occipital N400 amplitude [19]. Ortiz et al. used a passive vibrotactile device to concentrate on the variations in spatial brain processing assessed by event-related potentials (ERP) in children with blindness. They found that early interpretation of tactile input in children with normal vision and those who are blind have different ways of communicating cross-modal information. Children who are blind have shorter reaction times, earlier latencies for cognitive (perceptual) event-related potentials, and, conversely, a weaker ability to determine the spatial direction of the stimulus. However, they are equally adept at identifying stimuli containing semantic information, such as letters [19]. Locomotion on slopes given visual, acoustic, and tactile information was used by Alexandre et al. [20]. Both auditory and tactile cues during climb had advantageous effects on blind children: sound cues lengthened steps, while tactile cues decreased gait deviation and task completion time.

**Table 2:** Clinical characteristics and outcomes of the included studies. SSD: Sensory substitution device

Study	Objectives	Stimuli	Main outcomes	ROBIN-I
Mascle et al., 2022 [12]	To examine three tactile illustration designs in two populations—sighted children who were blindfolded and blind children.	The team made three lists of 10 French words. Only three items were allowed: animals, graspable objects, and plants.	Circles with texture might be recognized as quickly and precisely as textured shapes. Therefore, it would seem that using textures as illustrations in books for kids with visual impairments is a good idea.	High
Vinter et al., 2020 [13]	To determine whether young blind children could recognize tactile images more accurately than was previously thought possible when the experimental environment resembled early blind children's normal ecological reading situations.	The participants were given title information or listened to the accompanying text before examining the textured tactile images, just as they would in a real-world reading environment.	The findings revealed that their naming scores were identical to those of sighted children of the same age and were higher than those previously reported at comparable ages. The sighted and blind children benefited from the knowledge given prior to exploration.	Moderate
Theurel et al., 2013 [14]	To investigate haptic detection of tactile images by young children who are blind.	Participants were shown 24 stimuli, eight tactile images depicted using three distinct approaches (textures, thermoforming, and raised lines).	Textures facilitate picture recognition regardless of user level, and early and frequent usage of tactile material increases haptic competency. Practical consequences for tactile picture design are examined.	Moderate
Withagen et al., 2010 [15]	From birth to age 12, congenitally blind children were tested for tactile functioning.	The Tactual Profile is a tool created to assess a child's blindness-related tactile skills in terms of tactile sensory, motor, and perceptual functioning.	Approximately 94% of the tactile tasks that congenitally blind youngsters experience daily are successfully completed by these children without any additional disabilities. There is a slight individual difference, though.	Moderate
D'angiulli and Waraich, 2002 [16]	To examine the nature of blind individuals’ tactile superiority and its implications for cross-modal plasticity, we compared the tactile performance of congenitally totally blind, low-vision, and sighted children on a raised-line picture identification test.	Individual assessments of the kids—blindfolded, if sighted—were made. First, participants were told to examine the two practice drawings until they could identify the objects (with the experimenter's help if necessary).	These findings imply that cross-modal plasticity in congenital blindness may be cognitively correlated with improved perceptual encoding and recognition memory.	High
Ortiz Alonso et al., 2015 [17]	Differences in early stages of tactile ERP temporal sequence (P100) in the cortical organization during passive tactile stimulation in children with blindness and controls	In order to concentrate on the variations in spatial brain processing assessed by event-related potentials (ERP) in children with blindness, we used a passive vibrotactile device.	Early interpretation of tactile input Children with normal vision and those who are blind have different ways of communicating cross-modal information. Children who are blind have shorter reaction times, earlier latencies for cognitive (perceptual) event-related potentials, and conversely, a weaker ability to determine the spatial direction of the stimulus. However, they are equally adept at identifying stimuli containing semantic information, such as letters.	Moderate
Shen et al., 2022 [18]	To investigate whether Chinese children with congenital and acquired blindness have trouble comprehending concrete notions using perceptual symbols.	Experiment 1 employed the word-pair matching paradigm. The word-card-pairing paradigm was employed in Experiment 2 to examine how tactile experiences help blind children process concrete concepts.	Blind students can still express knowledge when the visual sensory channel is blocked by activating the spatial information of referents through the tactile sensory channel and the blind students' knowledge also includes knowledge that was acquired through other channels, primarily the tactile sensory channel.	Moderate
Ortiz et al., 2019 [19]	To enable high-level processing of tactile semantic content, we used long-term haptic tactile stimulation training with an SSD.	When the SSD touches the dominant hand, it turns images into a stimulation matrix. Children were asked to name letters, characterize line orientation, and read words.	For six months, subjects attended twice-daily training sessions five days per week. Children who are blind are better than controls at learning to recognise SSD-delivered letters, and they have a larger occipital N400 amplitude.	Moderate
Alexandre et al., 2012 [20]	To determine how children's locomotor modes, gait patterns, gait deviation, and time to ascend or descend various slopes were influenced by visual information, the degree of slope, and external auditory and tactile signals.	Locomotion on Slopes Given Visual, Acoustic, and Tactile Information	Both auditory and tactile cues during climb had advantageous effects on blind children: sound cues lengthened steps, while tactile cues decreased gait deviation and task completion time. Children who were blindfolded had considerably longer step lengths during descents in the sound cue condition compared to the tactile cue condition, and both sound and tactile cues lengthened the task's completion time.	High

Discussion

The aim of this systematic review was to study the tactile functioning perception among blind children from a clinical perspective. Up to the authors’ knowledge, this is the first review to investigate this object. Only one systematic review and meta-analysis discussed the effects of exercise programs on the balance of blind children and adolescents [21].

Six studies in this review reported that textured graphical objects, images, drawings, and illustrations were used as stimuli to test tactile functioning in blind children [12-14, 16, 19]. Textures facilitate picture recognition regardless of user level, and early and frequent usage of tactile material increases haptic competency. Practical consequences for tactile picture design are examined [14]. This combination of several textures might have improved how the viewer perceived the various pieces of the image, allowing for a greater ability to distinguish between the important aspects of the object. This would be in line with the findings of Morrongiello et al., who demonstrated that critical parts (such as the handle of a cup) played a role in object identification, especially in older children, and that children's representation of objects changed with increasing age from one based primarily on global shape to one that incorporates specific local parts of objects [22].

The complexity of the graphics may also be a factor in the variation in identification rates as a function of the illustration technique. Additionally, Lebaz demonstrated that tactual complexity had a stronger correlation with identification success than visual complexity. In fact, the sequential processing results in a working memory overload in the haptic system. The volume of information that must be retrieved thus appears to be a difficult issue [23].

Subjects participated in twice-daily training sessions for six months, five days a week. Blind children learn to recognize SSD-delivered letters more quickly than controls and have a greater occipital N400 amplitude [19]. In order to focus on the differences in spatial brain processing measured by ERP in children with blindness, Ortiz et al. used a passive vibrotactile device. They discovered that toddlers with normal vision and those who are blind have different ways of exchanging cross-modal information during the early interpretation of tactile input. Blind children react more quickly, experience cognitive (perceptual) event-related potentials earlier, and, on the other hand, have a reduced sense of the spatial orientation of the stimulus [19].

Without a doubt, naming tactile images is challenging when naive-sighted participants attempt so through touch [24]. Both congenitally blind and sighted subjects wearing blindfolds performed at extremely low levels when asked to name pictures with raised lines that represented common things. Although they performed better than the other participants, the adventitiously (late) blind nonetheless fared poorly. The advantage of the late blind (those who go blind after the first year of life) was attributed to their stronger tactile abilities and prior exposure to images.

This study reported that locomotion on slopes given visual, acoustic, and tactile information was used by Alexandre et al. [20]. Both auditory and tactile cues during climb had advantageous effects on blind children: sound cues lengthened steps, while tactile cues decreased gait deviation and task completion time. Children altered their locomotor styles to slope differences as was to be predicted. Similar to other studies [25], steeper slopes were more frequently used for the exploration of alternate forms of locomotion. Most kids considered slopes steeper than 20 degrees to be "non-walkable," and most of them opted to crawl up and slide down the hardest slopes. Falling suggests a faulty understanding of the slope's advantages. Children trip and fall because they overestimate their abilities and think that they can walk on a too-steep slope since they have good postural control. According to Plumert, children who tend to overestimate their skills are more likely to have accidents when they are 6 years old [26].

## Conclusions

The results of this systematic review demonstrated that textured images, words, and devices had a positive effect as tactile stimuli for blind children. It has been demonstrated that category information, familiarity, and complexity all affect how simple or difficult picture identification is. Blind persons can use pictorial displays efficiently, but they can benefit from instruction when foreshortening is used in complicated representations of three-dimensional things.
